# 5-(4-Fluoro­benzyl­idene)-4′-(4-fluoro­phen­yl)-1,1′-dimethyl­dispiro­[piperidine-3,3′-pyrrolidine-2′,3′′-indoline]-4,2′′-dione

**DOI:** 10.1107/S1600536812037245

**Published:** 2012-09-05

**Authors:** V. Rajni Swamy, S. Ramaswamy, N. Srinivasan, R. Ranjith Kumar, R.V. Krishnakumar

**Affiliations:** a Department of Physics, Thiagarajar College, Madurai 625 009, India; b Department of Physics, N.M.S.S. Vellaichamy Nadar College, Madurai 625 019, India; c School of Chemistry, Madurai Kamaraj University, Madurai 625 021, India

## Abstract

The asymmetric unit of the title compound, C_30_H_27_F_2_N_3_O_2_, contains two independent mol­ecules. The pyrrolidine five-membered ring assumes an envelope conformation (with the CH_2_ atom at the flap) in one mol­ecule and a twisted conformation in the other one. In both independent mol­ecules, the 4-piperidinone rings adopt a similar twisted chair conformation. In the crystal, the two independent mol­ecules form an *R*_2_^2^(8) dimer through a pair of N—H⋯O hydrogen bonds; the *R*_2_^2^(8) dimers are connected *via* weak C—H⋯O hydrogen bonds, leading to a chain extending along the *c* axis.

## Related literature

For background to and potential applications of the title compound, see: Baroudi *et al.* (1996 ▶); Dimmock *et al.* (2001 ▶); Hansch & Leo (1979 ▶); Hilton *et al.* (2000 ▶); Mobio *et al.* (1989 ▶); Mogilaiah & Rao (1998 ▶); Perumal *et al.* (2001 ▶); Sundberg (1996 ▶); Taft (1956 ▶); Watson *et al.* (2001 ▶). For hydrogen-bond motifs, see: Bernstein *et al.* (1995 ▶). For ring conformations based on puckering parameters, see: Cremer & Pople (1975 ▶).
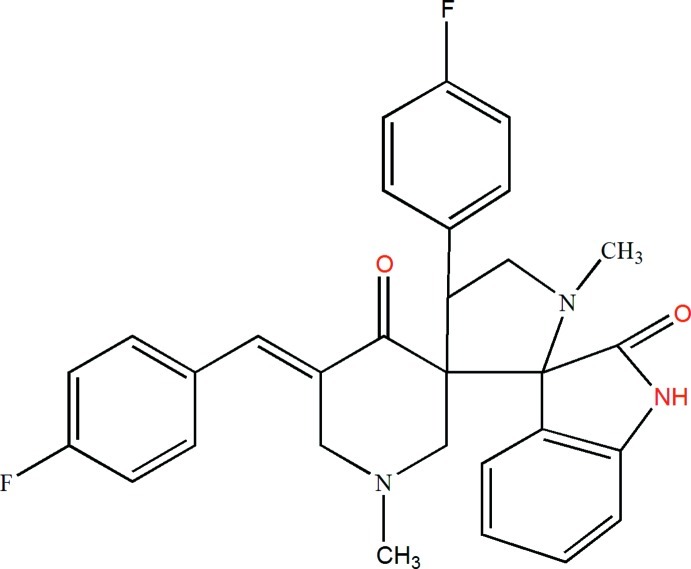


## Experimental

### 

#### Crystal data

C_30_H_27_F_2_N_3_O_2_
*M* *_r_* = 499.55
Monoclinic, 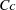

*a* = 27.1839 (15) Å
*b* = 10.2809 (5) Å
*c* = 18.7045 (11) Å
β = 107.92 (1)°
*V* = 4973.9 (5) Å^3^
*Z* = 8
Mo *K*α radiation
μ = 0.10 mm^−1^
*T* = 300 K
0.30 × 0.17 × 0.10 mm

#### Data collection

Bruker Kappa APEXII diffractometer
Absorption correction: multi-scan (*SADABS*; Sheldrick, 2004 ▶) *T*_min_ = 0.98, *T*_max_ = 0.99
32062 measured reflections
7610 independent reflections
5922 reflections with *I* > 2σ(*I*)
*R*_int_ = 0.024

#### Refinement

*R*[*F* ^2^ > 2σ(*F* ^2^)] = 0.038
*wR*(*F* ^2^) = 0.100
*S* = 1.04
7610 reflections
671 parameters
2 restraints
H-atom parameters constrained
Δρ_max_ = 0.17 e Å^−3^
Δρ_min_ = −0.17 e Å^−3^

### 

Data collection: *APEX2* (Bruker, 2004 ▶); cell refinement: *SAINT* (Bruker, 2004 ▶); data reduction: *SAINT*; program(s) used to solve structure: *SHELXS97* (Sheldrick, 2008 ▶); program(s) used to refine structure: *SHELXL97* (Sheldrick, 2008 ▶); molecular graphics: *PLATON* (Spek, 2003 ▶); software used to prepare material for publication: *SHELXL97*.

## Supplementary Material

Crystal structure: contains datablock(s) I, global. DOI: 10.1107/S1600536812037245/xu5607sup1.cif

Structure factors: contains datablock(s) I. DOI: 10.1107/S1600536812037245/xu5607Isup2.hkl

Additional supplementary materials:  crystallographic information; 3D view; checkCIF report

## Figures and Tables

**Table 1:** Hydrogen-bond geometry (Å, °)

*D*—H⋯*A*	*D*—H	H⋯*A*	*D*⋯*A*	*D*—H⋯*A*
N2*A*—H2*A*⋯O1*B*	0.86	2.11	2.959 (2)	169
N2*B*—H2*B*⋯O1*A*	0.86	2.14	2.984 (2)	169
C27*B*—H27*B*⋯O2*B* ^i^	0.93	2.49	3.314 (3)	147

Symmetry code: (i) 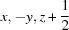.

**Fig. 1.**
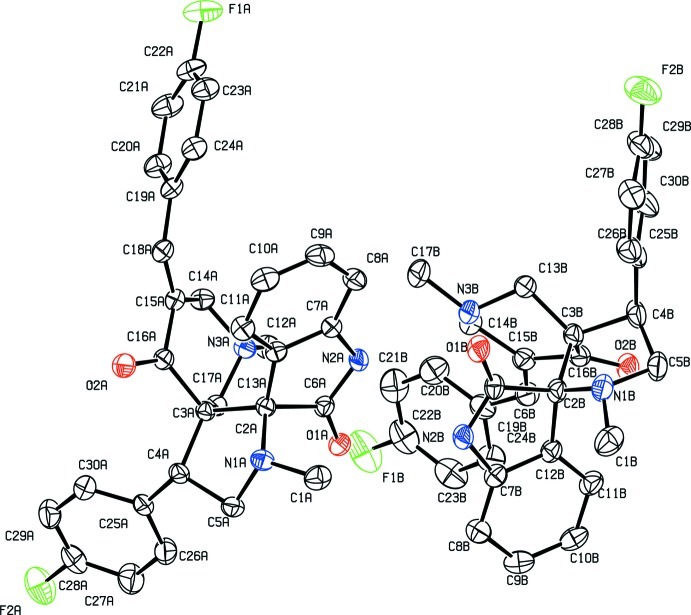
The molecular structure of (I), with atom labels and 30% probability displacement ellipsoids for non-H atoms.

**Fig. 2.**
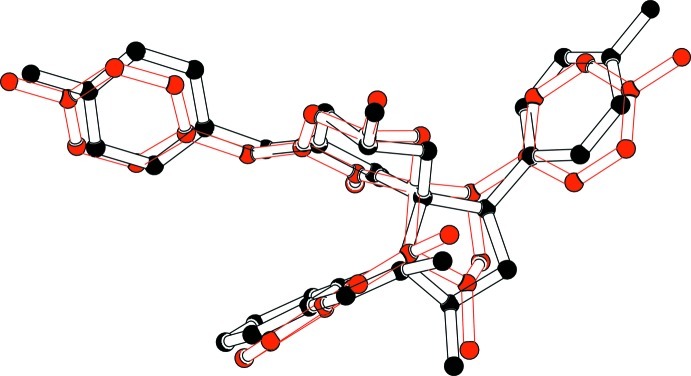
Overlay diagram of the two molecules A and B in the asymmetric unit showing differences in their conformation.

**Fig. 3.**
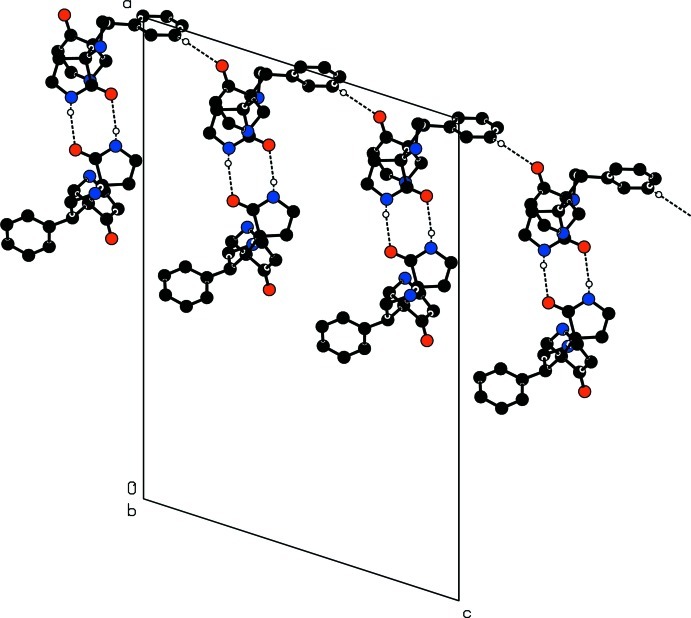
R22(8) dimers connected through C—H···O hydrogen bond leading to a chain along the c-axis. Non-participating rings, methyl groups and F atoms are omitted for clarity.

**Fig. 4.**
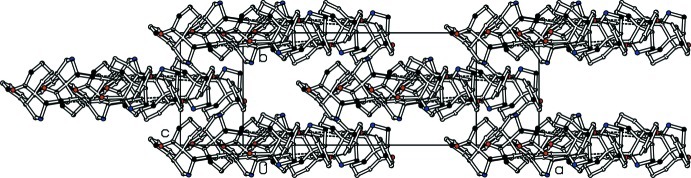
The packing of (I), viewed down the c axis. Non-participating rings, methyl groups and F atoms are omitted for clarity.

## supplementary crystallographic information

### Comment

The title compound may be regarded as a 'heterosystem' comprising of three distinct classes of heterocycles *viz*. oxindole, piperidine and pyrrolo ring systems each of which exhibits unique pharmacological properties. Compounds with an indole framework are reported to be promising pharmacophores (Hilton *et al.*, 2000) in addition to their antimicrobial and antitumor activities (Sundberg, 1996). Derivatives of 2,3-dihydro-2,3-dioxindole were reported as antibacterial (Mogilaiah & Rao, 1998), antifungal & antiviral (Hansch & Leo, 1979), antioxidant & cytotoxic agents (Taft, 1956). Piperidin-4-ones are reported to possess analgesic, anti-inflammatory, anti-cancer, anti-microbial activities and herbicidal properties (Mobio *et al.*, 1989; Dimmock *et al.*, 2001; Perumal *et al.*, 2001) and act as potential inhibitors of human placental aromatase in vitro (Baroudi *et al.*, 1996). Similarly, the pyrrolidine compounds have been reported to be potential inhibitors of diabetes, cancer and infection causing pathogens (Watson *et al.*, 2001). The present structural characterization using single-crystal X-ray intensity data was carried out with a view to understand drug design mechanisms and explore the possibility of discovery of a 'unidrug' with 'multiactivity' properties.

The title compound, C_33_H_27_N_3_O_2_F_2_ (I), crystallizes in the space group *Cc* with two molecules in the asymmetric unit. The two crystallographically independent molecules in the asymmetric unit differ significantly in their conformation as shown in the overlay diagram (Fig.2). Compound (I) is a di-spiro ring compound in which the spiro junction links the methyl piperidinone, the oxindole and methyl pyrrolidine rings. The conformation of the 5-membered methyl substituted pyrrolidine ring (N1—C2—C3—C4—C5) is envelope on C5A in molecule A and a twisted envelop about C5B and N1B in molecule B (Cremer & Pople, 1975). The corresponding puckering parameters of the pyrrolidine ring for molecules A and B are Q = 0.435 (2) Å, φ = 152.3 (3)° and Q = 0.411 (3) Å, φ = 338.3 (4)°, respectively. The piperidinone ring (N3—C13—C3—C16—C15—C14) adopts a slightly twisted chair conformation in both molecules with the puckering parameters of Q = 0.541 (2) Å, θ = 27.8 (2) ° and φ = 42.2 (5) ° in molecule A, Q = 0.537 (2) Å, θ = 33.9 (2)° and φ= 47.2 (5) ° in molecule B, respectively. The twist of the 4-fluorophenyl with respect to the spiro junction denoted by the torsion C3—C4—C25—C30 differs by about 10° in molecules A and B and that of the 4-fluorobenzylidene by about 20°.

The two crystallographically independent molecules in the asymmetric unit form an asymmetric R^2^_2_(8) (Bernstein *et al.*, 1995) dimer through strong N—H···O hydrogen bonds (N2A—H2A···O1B and N2B—H2B···O1A ) in which the N and carbonyl O1 of the pyrrolidine participates as donor and acceptor, respectively (Table1). The carbonyl O2 of molecule B participates in a C—H···O hydrogen bond [C27B—H27B···O2B (*x*, -*y*, *z* + 1/2)] which connects the*R*^2^_2_(8) dimers along the c-axis (Fig.3). These chains together with the C-centering of the lattice leads to packing of molecules as shown in Fig.4. Also, a potential solvent accessible void of volume 72.4Å^3^ which constitutes about 1.5% of the total unit cell volume is present.

### Experimental

A mixture of 1-methyl-3,5-bis[(*E*)-(4-fluorophenyl)methylidene]tetrahydro-\4(1*H*)-pyridinone (1 mmol), isatin (1 mmol) and sarcosine were dissolved in methanol (10 mL) and refluxed for 30 min. After completion of the reaction as evident from TLC, the mixture was poured into water (50 mL). The precipitated solid was filtered, washed with water and recrystallized from ethyl acetate to obtain pure 5-(4-fluorobenzylidene)-4'-(4-fluorophenyl)-1,1'-dimethyldispiro[piperidine-3,3'-pyrrolidine-2',3''-indoline]-4,2''-dione

### Refinement

H atoms bonded to N were located in a difference map and refined isotropically. Other H atoms were positioned geometrically and refined using a riding model, with C—H = 0.95–0.99 Å and with *U*_iso_(H) = 1.2 (1.5 for methyl groups) times *U*_eq_(C).

### Crystal data

**Table:**

C_30_H_27_F_2_N_3_O_2_	*F*(000) = 2096
*M**_r_* = 499.55	*D*_x_ = 1.334 Mg m^−^^3^
Monoclinic, *C**c*	Mo *K*α radiation, λ = 0.71073 Å
Hall symbol: C -2yc	Cell parameters from 5446 reflections
*a* = 27.1839 (15) Å	θ = 2–30°
*b* = 10.2809 (5) Å	µ = 0.10 mm^−^^1^
*c* = 18.7045 (11) Å	*T* = 300 K
β = 107.92 (1)°	Needle, yellow
*V* = 4973.9 (5) Å^3^	0.30 × 0.17 × 0.10 mm
*Z* = 8	

### Data collection

**Table:**

Bruker Kappa APEXII diffractometer	7610 independent reflections
Radiation source: fine-focus sealed tube	5922 reflections with *I* > 2σ(*I*)
Graphite monochromator	*R*_int_ = 0.024
ω and φ scan	θ_max_ = 30.6°, θ_min_ = 2.1°
Absorption correction: multi-scan (*SADABS*; Sheldrick, 2004)	*h* = −37→38
*T*_min_ = 0.98, *T*_max_ = 0.99	*k* = −14→14
32062 measured reflections	*l* = −26→26

### Refinement

**Table:**

Refinement on *F*^2^	Primary atom site location: structure-invariant direct methods
Least-squares matrix: full	Secondary atom site location: difference Fourier map
*R*[*F*^2^ > 2σ(*F*^2^)] = 0.038	Hydrogen site location: inferred from neighbouring sites
*wR*(*F*^2^) = 0.100	H-atom parameters constrained
*S* = 1.04	*w* = 1/[σ^2^(*F*_o_^2^) + (0.0514*P*)^2^ + 0.6706*P*] where *P* = (*F*_o_^2^ + 2*F*_c_^2^)/3
7610 reflections	(Δ/σ)_max_ < 0.001
671 parameters	Δρ_max_ = 0.17 e Å^−^^3^
2 restraints	Δρ_min_ = −0.17 e Å^−^^3^

### Special details

**Table:**

Geometry. All esds (except the esd in the dihedral angle between two l.s. planes) are estimated using the full covariance matrix. The cell esds are taken into account individually in the estimation of esds in distances, angles and torsion angles; correlations between esds in cell parameters are only used when they are defined by crystal symmetry. An approximate (isotropic) treatment of cell esds is used for estimating esds involving l.s. planes.
Refinement. Refinement of *F*^2^ against ALL reflections. The weighted *R*-factor *wR* and goodness of fit *S* are based on *F*^2^, conventional *R*-factors *R* are based on *F*, with *F* set to zero for negative *F*^2^. The threshold expression of *F*^2^ > σ(*F*^2^) is used only for calculating *R*-factors(gt) *etc*. and is not relevant to the choice of reflections for refinement. *R*-factors based on *F*^2^ are statistically about twice as large as those based on *F*, and *R*- factors based on ALL data will be even larger.

### Fractional atomic coordinates and isotropic or equivalent isotropic displacement parameters (Å^2^)

**Table:**

	*x*	*y*	*z*	*U*_iso_*/*U*_eq_	
F1A	0.19215 (9)	0.56542 (17)	0.68191 (10)	0.0879 (6)	
F2A	−0.09339 (8)	0.0793 (2)	0.00530 (12)	0.0990 (6)	
O1A	0.17862 (6)	−0.11493 (15)	0.28433 (8)	0.0446 (3)	
O2A	0.01866 (6)	−0.00921 (17)	0.39904 (9)	0.0527 (4)	
N1A	0.10157 (7)	−0.26369 (15)	0.34659 (10)	0.0414 (4)	
N2A	0.21349 (6)	−0.09677 (17)	0.41157 (9)	0.0412 (4)	
H2A	0.2451	−0.0840	0.4131	0.049*	
N3A	0.13347 (6)	0.14582 (16)	0.32927 (9)	0.0384 (3)	
C1A	0.13876 (10)	−0.3696 (2)	0.36108 (14)	0.0547 (5)	
H87A	0.1582	−0.3710	0.4135	0.082*	
H88A	0.1619	−0.3571	0.3319	0.082*	
H89A	0.1208	−0.4507	0.3474	0.082*	
C2A	0.12304 (7)	−0.13338 (17)	0.36896 (10)	0.0340 (3)	
C3A	0.07630 (7)	−0.03987 (17)	0.32532 (10)	0.0335 (3)	
C4A	0.03564 (7)	−0.13532 (19)	0.27477 (11)	0.0389 (4)	
H4A	0.0126	−0.1609	0.3036	0.047*	
C5A	0.06726 (8)	−0.2543 (2)	0.26972 (12)	0.0433 (4)	
H91A	0.0457	−0.3310	0.2555	0.052*	
H92A	0.0865	−0.2414	0.2343	0.052*	
C6A	0.17391 (7)	−0.11003 (18)	0.34750 (11)	0.0367 (4)	
C7A	0.19686 (7)	−0.10634 (18)	0.47541 (11)	0.0379 (4)	
C8A	0.22660 (9)	−0.1054 (2)	0.54990 (13)	0.0498 (5)	
H8A	0.2623	−0.0950	0.5638	0.060*	
C9A	0.20116 (11)	−0.1208 (2)	0.60309 (13)	0.0561 (6)	
H9A	0.2200	−0.1179	0.6538	0.067*	
C10A	0.14876 (11)	−0.1400 (2)	0.58260 (13)	0.0527 (5)	
H10A	0.1327	−0.1503	0.6196	0.063*	
C11A	0.11948 (9)	−0.1445 (2)	0.50774 (12)	0.0439 (4)	
H11A	0.0841	−0.1602	0.4940	0.053*	
C12A	0.14382 (7)	−0.12508 (17)	0.45357 (11)	0.0359 (4)	
C13A	0.09296 (7)	0.06559 (18)	0.27962 (10)	0.0359 (4)	
H13A	0.1057	0.0253	0.2418	0.043*	
H13B	0.0635	0.1195	0.2542	0.043*	
C14A	0.11215 (9)	0.22787 (19)	0.37561 (11)	0.0424 (4)	
H14A	0.0882	0.2895	0.3439	0.051*	
H14B	0.1399	0.2768	0.4103	0.051*	
C15A	0.08455 (7)	0.14907 (18)	0.41932 (11)	0.0374 (4)	
C16A	0.05583 (7)	0.03020 (18)	0.38291 (11)	0.0373 (4)	
C17A	0.15762 (10)	0.2274 (3)	0.28577 (14)	0.0547 (6)	
H17A	0.1813	0.2866	0.3188	0.082*	
H17B	0.1315	0.2757	0.2492	0.082*	
H17C	0.1760	0.1735	0.2607	0.082*	
C18A	0.08585 (8)	0.17374 (19)	0.48987 (12)	0.0415 (4)	
H18A	0.0675	0.1165	0.5105	0.050*	
C19A	0.11260 (8)	0.27963 (19)	0.53886 (11)	0.0412 (4)	
C20A	0.11264 (10)	0.4074 (2)	0.51427 (13)	0.0541 (5)	
H20A	0.0948	0.4281	0.4646	0.065*	
C21A	0.13879 (13)	0.5039 (2)	0.56249 (16)	0.0638 (7)	
H21A	0.1384	0.5893	0.5458	0.077*	
C22A	0.16499 (11)	0.4720 (2)	0.63464 (15)	0.0592 (6)	
C23A	0.16558 (10)	0.3486 (2)	0.66213 (14)	0.0548 (5)	
H23A	0.1837	0.3295	0.7119	0.066*	
C24A	0.13846 (9)	0.2529 (2)	0.61406 (12)	0.0463 (5)	
H24A	0.1375	0.1691	0.6323	0.056*	
C25A	0.00183 (7)	−0.0801 (2)	0.20117 (12)	0.0429 (4)	
C26A	0.00969 (10)	−0.1043 (3)	0.13277 (14)	0.0629 (7)	
H26A	0.0370	−0.1575	0.1310	0.075*	
C27A	−0.02242 (12)	−0.0506 (4)	0.06644 (17)	0.0778 (9)	
H27A	−0.0168	−0.0674	0.0207	0.093*	
C28A	−0.06194 (10)	0.0267 (3)	0.06989 (17)	0.0659 (7)	
C29A	−0.07170 (10)	0.0522 (3)	0.13582 (18)	0.0666 (7)	
H29A	−0.0992	0.1053	0.1369	0.080*	
C30A	−0.03971 (9)	−0.0029 (3)	0.20095 (14)	0.0565 (6)	
H30A	−0.0464	0.0126	0.2461	0.068*	
F1B	0.23620 (8)	0.5490 (2)	−0.06501 (14)	0.1029 (7)	
F2B	0.52298 (9)	0.1231 (3)	0.69694 (10)	0.1016 (7)	
O1B	0.31860 (6)	−0.07362 (18)	0.39742 (9)	0.0531 (4)	
O2B	0.45137 (6)	0.10897 (18)	0.24933 (10)	0.0561 (4)	
N1B	0.40951 (8)	−0.1761 (2)	0.36301 (11)	0.0538 (5)	
N2B	0.28524 (7)	−0.10658 (19)	0.27116 (10)	0.0459 (4)	
H2B	0.2532	−0.1127	0.2686	0.055*	
N3B	0.33289 (7)	0.1840 (2)	0.33054 (11)	0.0514 (4)	
C1B	0.39641 (13)	−0.3102 (3)	0.34123 (18)	0.0742 (8)	
H87B	0.4193	−0.3671	0.3770	0.111*	
H88B	0.3999	−0.3253	0.2924	0.111*	
H89B	0.3614	−0.3269	0.3399	0.111*	
C2B	0.37587 (8)	−0.0769 (2)	0.31609 (12)	0.0435 (4)	
C3B	0.40885 (8)	0.0528 (2)	0.34003 (12)	0.0446 (4)	
C4B	0.46372 (8)	0.0044 (3)	0.38855 (13)	0.0554 (6)	
H4B	0.4895	0.0459	0.3691	0.066*	
C5B	0.46256 (9)	−0.1388 (3)	0.37037 (16)	0.0633 (7)	
H91B	0.4705	−0.1536	0.3239	0.076*	
H92B	0.4869	−0.1868	0.4105	0.076*	
C6B	0.32370 (8)	−0.0802 (2)	0.33523 (12)	0.0428 (4)	
C7B	0.30374 (8)	−0.12272 (19)	0.20954 (12)	0.0431 (4)	
C8B	0.27583 (10)	−0.1550 (2)	0.13696 (14)	0.0532 (5)	
H8B	0.2403	−0.1678	0.1231	0.064*	
C9B	0.30287 (11)	−0.1676 (3)	0.08526 (14)	0.0606 (6)	
H9B	0.2850	−0.1884	0.0357	0.073*	
C10B	0.35529 (11)	−0.1499 (2)	0.10606 (14)	0.0578 (6)	
H10B	0.3724	−0.1573	0.0702	0.069*	
C11B	0.38315 (10)	−0.1212 (2)	0.17991 (14)	0.0516 (5)	
H11B	0.4189	−0.1126	0.1941	0.062*	
C12B	0.35699 (8)	−0.1056 (2)	0.23217 (12)	0.0425 (4)	
C13B	0.38366 (8)	0.1486 (2)	0.38058 (13)	0.0499 (5)	
H13C	0.3803	0.1090	0.4259	0.060*	
H13D	0.4050	0.2257	0.3947	0.060*	
C14B	0.33674 (9)	0.2680 (2)	0.27046 (14)	0.0543 (5)	
H14C	0.3501	0.3519	0.2913	0.065*	
H14D	0.3025	0.2817	0.2355	0.065*	
C15B	0.37126 (8)	0.2129 (2)	0.22862 (13)	0.0476 (5)	
C16B	0.41365 (8)	0.1241 (2)	0.26963 (12)	0.0448 (4)	
C17B	0.30194 (11)	0.2453 (4)	0.37246 (17)	0.0756 (9)	
H17D	0.2959	0.1841	0.4076	0.113*	
H17E	0.2695	0.2729	0.3382	0.113*	
H17F	0.3201	0.3194	0.3992	0.113*	
C18B	0.36800 (9)	0.2394 (2)	0.15730 (14)	0.0513 (5)	
H18B	0.3924	0.1970	0.1402	0.062*	
C19B	0.33263 (9)	0.3235 (2)	0.10158 (14)	0.0542 (5)	
C20B	0.30487 (12)	0.4260 (3)	0.11879 (19)	0.0685 (7)	
H20B	0.3083	0.4443	0.1688	0.082*	
C21B	0.27218 (12)	0.5015 (3)	0.0624 (2)	0.0763 (8)	
H21B	0.2533	0.5692	0.0741	0.092*	
C22B	0.26832 (12)	0.4744 (3)	−0.0100 (2)	0.0744 (8)	
C23B	0.29484 (13)	0.3763 (3)	−0.02990 (18)	0.0757 (8)	
H23B	0.2911	0.3599	−0.0802	0.091*	
C24B	0.32757 (11)	0.3014 (3)	0.02611 (16)	0.0633 (6)	
H24B	0.3465	0.2351	0.0132	0.076*	
C25B	0.47780 (8)	0.0347 (3)	0.47180 (13)	0.0541 (5)	
C26B	0.46798 (10)	−0.0497 (3)	0.52376 (15)	0.0590 (6)	
H26B	0.4506	−0.1273	0.5073	0.071*	
C27B	0.48357 (11)	−0.0204 (3)	0.59932 (15)	0.0667 (7)	
H27B	0.4775	−0.0782	0.6339	0.080*	
C28B	0.50786 (10)	0.0944 (4)	0.62228 (15)	0.0691 (8)	
C29B	0.51688 (12)	0.1823 (4)	0.57389 (17)	0.0787 (9)	
H29B	0.5327	0.2615	0.5910	0.094*	
C30B	0.50187 (11)	0.1510 (3)	0.49813 (16)	0.0718 (8)	
H30B	0.5082	0.2099	0.4642	0.086*	

### Atomic displacement parameters (Å^2^)

**Table:**

	*U*^11^	*U*^22^	*U*^33^	*U*^12^	*U*^13^	*U*^23^
F1A	0.1272 (16)	0.0666 (10)	0.0707 (11)	−0.0240 (10)	0.0319 (11)	−0.0296 (8)
F2A	0.0756 (12)	0.1189 (16)	0.0805 (13)	0.0201 (11)	−0.0081 (10)	0.0300 (11)
O1A	0.0422 (7)	0.0589 (8)	0.0386 (8)	−0.0014 (6)	0.0211 (6)	−0.0053 (6)
O2A	0.0449 (8)	0.0683 (10)	0.0548 (9)	−0.0129 (7)	0.0296 (7)	−0.0121 (7)
N1A	0.0444 (8)	0.0374 (8)	0.0406 (9)	−0.0054 (6)	0.0102 (7)	−0.0013 (6)
N2A	0.0312 (8)	0.0543 (9)	0.0401 (9)	−0.0006 (6)	0.0136 (7)	−0.0040 (7)
N3A	0.0374 (8)	0.0460 (8)	0.0335 (8)	−0.0081 (6)	0.0135 (6)	−0.0014 (6)
C1A	0.0669 (15)	0.0407 (10)	0.0546 (13)	0.0054 (10)	0.0159 (11)	0.0027 (9)
C2A	0.0325 (8)	0.0389 (8)	0.0323 (9)	−0.0034 (6)	0.0124 (7)	−0.0007 (7)
C3A	0.0298 (8)	0.0402 (8)	0.0312 (8)	−0.0036 (6)	0.0105 (7)	−0.0027 (7)
C4A	0.0315 (8)	0.0473 (9)	0.0387 (10)	−0.0080 (7)	0.0120 (7)	−0.0065 (8)
C5A	0.0426 (10)	0.0434 (9)	0.0432 (11)	−0.0067 (8)	0.0121 (8)	−0.0089 (8)
C6A	0.0355 (9)	0.0388 (9)	0.0393 (10)	−0.0003 (7)	0.0164 (8)	−0.0023 (7)
C7A	0.0377 (9)	0.0387 (9)	0.0380 (10)	0.0005 (7)	0.0126 (8)	−0.0018 (7)
C8A	0.0457 (11)	0.0538 (12)	0.0426 (11)	0.0024 (9)	0.0028 (9)	−0.0051 (9)
C9A	0.0715 (16)	0.0566 (13)	0.0329 (11)	0.0015 (11)	0.0053 (10)	−0.0009 (9)
C10A	0.0726 (15)	0.0531 (12)	0.0378 (11)	−0.0006 (10)	0.0247 (11)	0.0046 (9)
C11A	0.0475 (11)	0.0478 (10)	0.0400 (11)	−0.0037 (8)	0.0188 (9)	0.0081 (8)
C12A	0.0401 (9)	0.0358 (8)	0.0336 (9)	−0.0010 (7)	0.0140 (7)	0.0009 (7)
C13A	0.0353 (9)	0.0435 (9)	0.0308 (9)	−0.0033 (7)	0.0129 (7)	0.0009 (7)
C14A	0.0505 (11)	0.0417 (9)	0.0374 (10)	−0.0054 (8)	0.0171 (8)	−0.0010 (8)
C15A	0.0369 (9)	0.0384 (8)	0.0393 (10)	0.0032 (7)	0.0152 (8)	0.0001 (7)
C16A	0.0351 (9)	0.0439 (9)	0.0351 (10)	−0.0013 (7)	0.0141 (7)	−0.0013 (7)
C17A	0.0561 (13)	0.0660 (14)	0.0489 (12)	−0.0229 (11)	0.0261 (10)	−0.0058 (10)
C18A	0.0440 (10)	0.0421 (9)	0.0433 (11)	0.0021 (8)	0.0208 (9)	0.0002 (8)
C19A	0.0488 (11)	0.0415 (9)	0.0389 (10)	0.0039 (8)	0.0216 (9)	−0.0034 (7)
C20A	0.0722 (15)	0.0448 (10)	0.0438 (12)	0.0121 (10)	0.0158 (11)	0.0027 (9)
C21A	0.0954 (19)	0.0377 (10)	0.0602 (15)	0.0047 (11)	0.0268 (14)	−0.0018 (10)
C22A	0.0792 (17)	0.0523 (12)	0.0535 (14)	−0.0053 (11)	0.0315 (13)	−0.0172 (10)
C23A	0.0677 (15)	0.0590 (13)	0.0392 (11)	0.0005 (11)	0.0187 (10)	−0.0032 (9)
C24A	0.0569 (12)	0.0456 (10)	0.0405 (11)	−0.0001 (9)	0.0208 (9)	0.0034 (8)
C25A	0.0313 (9)	0.0516 (11)	0.0443 (11)	−0.0062 (7)	0.0096 (8)	−0.0083 (8)
C26A	0.0464 (12)	0.097 (2)	0.0432 (13)	0.0168 (12)	0.0111 (10)	−0.0057 (12)
C27A	0.0623 (16)	0.122 (3)	0.0453 (14)	0.0205 (16)	0.0115 (12)	0.0038 (15)
C28A	0.0465 (13)	0.0729 (16)	0.0645 (17)	−0.0024 (11)	−0.0033 (12)	0.0090 (13)
C29A	0.0453 (12)	0.0672 (15)	0.081 (2)	0.0126 (11)	0.0104 (12)	−0.0005 (13)
C30A	0.0474 (12)	0.0651 (14)	0.0567 (14)	0.0083 (10)	0.0158 (11)	−0.0101 (11)
F1B	0.0797 (12)	0.0951 (14)	0.1190 (16)	−0.0052 (10)	0.0085 (11)	0.0524 (12)
F2B	0.0958 (14)	0.154 (2)	0.0515 (10)	−0.0293 (14)	0.0179 (9)	−0.0204 (11)
O1B	0.0450 (8)	0.0760 (10)	0.0461 (9)	0.0011 (7)	0.0255 (7)	0.0027 (7)
O2B	0.0418 (8)	0.0777 (11)	0.0564 (10)	−0.0021 (7)	0.0262 (7)	0.0008 (8)
N1B	0.0469 (10)	0.0654 (12)	0.0517 (11)	0.0139 (8)	0.0192 (8)	0.0039 (9)
N2B	0.0351 (8)	0.0592 (10)	0.0487 (10)	−0.0029 (7)	0.0207 (8)	−0.0007 (8)
N3B	0.0393 (9)	0.0671 (11)	0.0513 (11)	0.0027 (8)	0.0189 (8)	−0.0114 (9)
C1B	0.088 (2)	0.0637 (15)	0.0734 (19)	0.0211 (14)	0.0277 (16)	0.0047 (14)
C2B	0.0350 (9)	0.0576 (11)	0.0429 (11)	0.0022 (8)	0.0196 (8)	−0.0009 (9)
C3B	0.0322 (9)	0.0624 (12)	0.0419 (11)	−0.0017 (8)	0.0152 (8)	−0.0047 (9)
C4B	0.0304 (10)	0.0909 (18)	0.0472 (12)	0.0035 (10)	0.0153 (9)	0.0034 (11)
C5B	0.0413 (12)	0.0947 (19)	0.0560 (14)	0.0187 (12)	0.0180 (11)	−0.0002 (13)
C6B	0.0363 (9)	0.0502 (10)	0.0470 (12)	0.0024 (8)	0.0206 (9)	0.0054 (8)
C7B	0.0470 (11)	0.0414 (9)	0.0445 (11)	0.0002 (8)	0.0195 (9)	0.0020 (8)
C8B	0.0513 (12)	0.0554 (12)	0.0512 (13)	−0.0040 (9)	0.0132 (10)	−0.0022 (10)
C9B	0.0790 (17)	0.0580 (13)	0.0456 (13)	−0.0062 (12)	0.0204 (12)	−0.0076 (10)
C10B	0.0793 (17)	0.0570 (13)	0.0484 (13)	0.0016 (12)	0.0364 (12)	−0.0053 (10)
C11B	0.0539 (13)	0.0558 (12)	0.0536 (13)	−0.0008 (9)	0.0289 (11)	−0.0058 (10)
C12B	0.0422 (10)	0.0462 (10)	0.0443 (11)	0.0007 (8)	0.0208 (9)	−0.0005 (8)
C13B	0.0399 (10)	0.0671 (13)	0.0448 (12)	−0.0028 (9)	0.0162 (9)	−0.0127 (10)
C14B	0.0465 (11)	0.0587 (13)	0.0576 (14)	0.0050 (9)	0.0160 (10)	−0.0095 (11)
C15B	0.0440 (11)	0.0489 (11)	0.0513 (12)	−0.0069 (8)	0.0166 (9)	−0.0082 (9)
C16B	0.0386 (10)	0.0538 (11)	0.0445 (11)	−0.0085 (8)	0.0165 (9)	−0.0077 (9)
C17B	0.0514 (14)	0.111 (2)	0.0695 (18)	0.0140 (14)	0.0254 (13)	−0.0220 (16)
C18B	0.0488 (11)	0.0533 (12)	0.0536 (13)	−0.0041 (9)	0.0183 (10)	−0.0054 (10)
C19B	0.0521 (13)	0.0520 (12)	0.0576 (14)	−0.0099 (10)	0.0156 (11)	−0.0006 (10)
C20B	0.0771 (18)	0.0548 (13)	0.0748 (18)	−0.0011 (12)	0.0251 (15)	0.0019 (12)
C21B	0.0718 (18)	0.0550 (14)	0.104 (3)	0.0025 (13)	0.0289 (17)	0.0157 (15)
C22B	0.0617 (16)	0.0692 (17)	0.086 (2)	−0.0140 (13)	0.0142 (15)	0.0271 (15)
C23B	0.0773 (19)	0.085 (2)	0.0599 (17)	−0.0169 (16)	0.0137 (15)	0.0127 (14)
C24B	0.0668 (16)	0.0644 (14)	0.0606 (15)	−0.0076 (12)	0.0227 (13)	0.0014 (12)
C25B	0.0308 (9)	0.0870 (16)	0.0443 (12)	−0.0026 (10)	0.0113 (9)	0.0033 (11)
C26B	0.0478 (13)	0.0768 (16)	0.0530 (14)	−0.0040 (11)	0.0164 (11)	0.0012 (11)
C27B	0.0557 (14)	0.097 (2)	0.0491 (14)	−0.0024 (14)	0.0187 (11)	0.0100 (13)
C28B	0.0470 (13)	0.112 (2)	0.0450 (13)	−0.0094 (14)	0.0096 (11)	−0.0077 (14)
C29B	0.0630 (17)	0.102 (2)	0.0640 (17)	−0.0295 (15)	0.0085 (14)	−0.0116 (16)
C30B	0.0529 (14)	0.103 (2)	0.0547 (15)	−0.0271 (14)	0.0100 (12)	0.0069 (14)

### Geometric parameters (Å, º)

**Table:**

F1A—C22A	1.360 (3)	F1B—C22B	1.363 (3)
F2A—C28A	1.359 (3)	F2B—C28B	1.361 (3)
O1A—C6A	1.228 (2)	O1B—C6B	1.215 (3)
O2A—C16A	1.210 (2)	O2B—C16B	1.208 (3)
N1A—C1A	1.454 (3)	N1B—C1B	1.451 (4)
N1A—C5A	1.457 (3)	N1B—C5B	1.457 (3)
N1A—C2A	1.470 (2)	N1B—C2B	1.468 (3)
N2A—C6A	1.349 (3)	N2B—C6B	1.353 (3)
N2A—C7A	1.404 (3)	N2B—C7B	1.402 (3)
N2A—H2A	0.8599	N2B—H2B	0.8601
N3A—C14A	1.452 (3)	N3B—C14B	1.446 (3)
N3A—C17A	1.458 (3)	N3B—C13B	1.455 (3)
N3A—C13A	1.458 (2)	N3B—C17B	1.458 (3)
C1A—H87A	0.9600	C1B—H87B	0.9600
C1A—H88A	0.9600	C1B—H88B	0.9600
C1A—H89A	0.9600	C1B—H89B	0.9600
C2A—C12A	1.510 (3)	C2B—C12B	1.523 (3)
C2A—C6A	1.572 (3)	C2B—C6B	1.566 (3)
C2A—C3A	1.602 (3)	C2B—C3B	1.591 (3)
C3A—C13A	1.533 (2)	C3B—C13B	1.528 (3)
C3A—C16A	1.536 (3)	C3B—C16B	1.547 (3)
C3A—C4A	1.561 (2)	C3B—C4B	1.569 (3)
C4A—C25A	1.511 (3)	C4B—C5B	1.510 (4)
C4A—C5A	1.515 (3)	C4B—C25B	1.517 (3)
C4A—H4A	0.9800	C4B—H4B	0.9800
C5A—H91A	0.9700	C5B—H91B	0.9700
C5A—H92A	0.9700	C5B—H92B	0.9700
C7A—C8A	1.379 (3)	C7B—C8B	1.377 (3)
C7A—C12A	1.386 (3)	C7B—C12B	1.389 (3)
C8A—C9A	1.384 (4)	C8B—C9B	1.389 (4)
C8A—H8A	0.9300	C8B—H8B	0.9300
C9A—C10A	1.371 (4)	C9B—C10B	1.369 (4)
C9A—H9A	0.9300	C9B—H9B	0.9300
C10A—C11A	1.382 (3)	C10B—C11B	1.388 (4)
C10A—H10A	0.9300	C10B—H10B	0.9300
C11A—C12A	1.385 (3)	C11B—C12B	1.384 (3)
C11A—H11A	0.9300	C11B—H11B	0.9300
C13A—H13A	0.9700	C13B—H13C	0.9700
C13A—H13B	0.9700	C13B—H13D	0.9700
C14A—C15A	1.505 (3)	C14B—C15B	1.505 (3)
C14A—H14A	0.9700	C14B—H14C	0.9700
C14A—H14B	0.9700	C14B—H14D	0.9700
C15A—C18A	1.333 (3)	C15B—C18B	1.337 (3)
C15A—C16A	1.497 (3)	C15B—C16B	1.485 (3)
C17A—H17A	0.9600	C17B—H17D	0.9600
C17A—H17B	0.9600	C17B—H17E	0.9600
C17A—H17C	0.9600	C17B—H17F	0.9600
C18A—C19A	1.464 (3)	C18B—C19B	1.464 (4)
C18A—H18A	0.9300	C18B—H18B	0.9300
C19A—C20A	1.392 (3)	C19B—C20B	1.391 (4)
C19A—C24A	1.393 (3)	C19B—C24B	1.394 (4)
C20A—C21A	1.382 (4)	C20B—C21B	1.387 (4)
C20A—H20A	0.9300	C20B—H20B	0.9300
C21A—C22A	1.358 (4)	C21B—C22B	1.356 (5)
C21A—H21A	0.9300	C21B—H21B	0.9300
C22A—C23A	1.367 (4)	C22B—C23B	1.356 (5)
C23A—C24A	1.382 (3)	C23B—C24B	1.382 (4)
C23A—H23A	0.9300	C23B—H23B	0.9300
C24A—H24A	0.9300	C24B—H24B	0.9300
C25A—C30A	1.380 (3)	C25B—C30B	1.380 (4)
C25A—C26A	1.383 (3)	C25B—C26B	1.388 (4)
C26A—C27A	1.393 (4)	C26B—C27B	1.378 (4)
C26A—H26A	0.9300	C26B—H26B	0.9300
C27A—C28A	1.353 (4)	C27B—C28B	1.356 (5)
C27A—H27A	0.9300	C27B—H27B	0.9300
C28A—C29A	1.364 (4)	C28B—C29B	1.354 (5)
C29A—C30A	1.382 (4)	C29B—C30B	1.387 (4)
C29A—H29A	0.9300	C29B—H29B	0.9300
C30A—H30A	0.9300	C30B—H30B	0.9300
			
C1A—N1A—C5A	115.54 (17)	C1B—N1B—C5B	115.3 (2)
C1A—N1A—C2A	115.94 (16)	C1B—N1B—C2B	116.0 (2)
C5A—N1A—C2A	107.36 (15)	C5B—N1B—C2B	106.87 (19)
C6A—N2A—C7A	111.77 (16)	C6B—N2B—C7B	112.13 (17)
C6A—N2A—H2A	124.1	C6B—N2B—H2B	123.9
C7A—N2A—H2A	124.1	C7B—N2B—H2B	124.0
C14A—N3A—C17A	109.27 (17)	C14B—N3B—C13B	111.60 (18)
C14A—N3A—C13A	110.30 (15)	C14B—N3B—C17B	110.6 (2)
C17A—N3A—C13A	110.58 (15)	C13B—N3B—C17B	110.6 (2)
N1A—C1A—H87A	109.5	N1B—C1B—H87B	109.5
N1A—C1A—H88A	109.5	N1B—C1B—H88B	109.5
H87A—C1A—H88A	109.5	H87B—C1B—H88B	109.5
N1A—C1A—H89A	109.5	N1B—C1B—H89B	109.5
H87A—C1A—H89A	109.5	H87B—C1B—H89B	109.5
H88A—C1A—H89A	109.5	H88B—C1B—H89B	109.5
N1A—C2A—C12A	109.73 (15)	N1B—C2B—C12B	115.60 (18)
N1A—C2A—C6A	111.70 (15)	N1B—C2B—C6B	106.82 (17)
C12A—C2A—C6A	100.61 (14)	C12B—C2B—C6B	100.93 (16)
N1A—C2A—C3A	102.64 (14)	N1B—C2B—C3B	102.73 (17)
C12A—C2A—C3A	118.84 (15)	C12B—C2B—C3B	115.74 (17)
C6A—C2A—C3A	113.58 (14)	C6B—C2B—C3B	115.19 (16)
C13A—C3A—C16A	107.00 (15)	C13B—C3B—C16B	106.55 (19)
C13A—C3A—C4A	112.50 (15)	C13B—C3B—C4B	114.11 (18)
C16A—C3A—C4A	112.21 (14)	C16B—C3B—C4B	109.15 (16)
C13A—C3A—C2A	112.62 (14)	C13B—C3B—C2B	112.33 (16)
C16A—C3A—C2A	109.02 (14)	C16B—C3B—C2B	110.19 (17)
C4A—C3A—C2A	103.54 (14)	C4B—C3B—C2B	104.51 (18)
C25A—C4A—C5A	116.37 (17)	C5B—C4B—C25B	114.3 (2)
C25A—C4A—C3A	115.76 (16)	C5B—C4B—C3B	103.19 (19)
C5A—C4A—C3A	103.65 (15)	C25B—C4B—C3B	115.05 (18)
C25A—C4A—H4A	106.8	C5B—C4B—H4B	108.0
C5A—C4A—H4A	106.8	C25B—C4B—H4B	108.0
C3A—C4A—H4A	106.8	C3B—C4B—H4B	108.0
N1A—C5A—C4A	101.25 (16)	N1B—C5B—C4B	103.27 (18)
N1A—C5A—H91A	111.5	N1B—C5B—H91B	111.1
C4A—C5A—H91A	111.5	C4B—C5B—H91B	111.1
N1A—C5A—H92A	111.5	N1B—C5B—H92B	111.1
C4A—C5A—H92A	111.5	C4B—C5B—H92B	111.1
H91A—C5A—H92A	109.3	H91B—C5B—H92B	109.1
O1A—C6A—N2A	124.62 (17)	O1B—C6B—N2B	125.06 (18)
O1A—C6A—C2A	126.84 (17)	O1B—C6B—C2B	126.55 (19)
N2A—C6A—C2A	108.21 (16)	N2B—C6B—C2B	108.02 (17)
C8A—C7A—C12A	122.15 (19)	C8B—C7B—C12B	122.5 (2)
C8A—C7A—N2A	128.06 (19)	C8B—C7B—N2B	127.7 (2)
C12A—C7A—N2A	109.70 (17)	C12B—C7B—N2B	109.78 (18)
C7A—C8A—C9A	117.3 (2)	C7B—C8B—C9B	117.4 (2)
C7A—C8A—H8A	121.4	C7B—C8B—H8B	121.3
C9A—C8A—H8A	121.4	C9B—C8B—H8B	121.3
C10A—C9A—C8A	121.4 (2)	C10B—C9B—C8B	121.2 (2)
C10A—C9A—H9A	119.3	C10B—C9B—H9B	119.4
C8A—C9A—H9A	119.3	C8B—C9B—H9B	119.4
C9A—C10A—C11A	120.9 (2)	C9B—C10B—C11B	120.9 (2)
C9A—C10A—H10A	119.6	C9B—C10B—H10B	119.6
C11A—C10A—H10A	119.6	C11B—C10B—H10B	119.6
C10A—C11A—C12A	118.7 (2)	C12B—C11B—C10B	119.1 (2)
C10A—C11A—H11A	120.6	C12B—C11B—H11B	120.5
C12A—C11A—H11A	120.6	C10B—C11B—H11B	120.5
C11A—C12A—C7A	119.50 (18)	C11B—C12B—C7B	118.9 (2)
C11A—C12A—C2A	130.53 (18)	C11B—C12B—C2B	131.9 (2)
C7A—C12A—C2A	109.67 (16)	C7B—C12B—C2B	109.14 (17)
N3A—C13A—C3A	109.87 (15)	N3B—C13B—C3B	108.85 (17)
N3A—C13A—H13A	109.7	N3B—C13B—H13C	109.9
C3A—C13A—H13A	109.7	C3B—C13B—H13C	109.9
N3A—C13A—H13B	109.7	N3B—C13B—H13D	109.9
C3A—C13A—H13B	109.7	C3B—C13B—H13D	109.9
H13A—C13A—H13B	108.2	H13C—C13B—H13D	108.3
N3A—C14A—C15A	111.64 (16)	N3B—C14B—C15B	112.63 (19)
N3A—C14A—H14A	109.3	N3B—C14B—H14C	109.1
C15A—C14A—H14A	109.3	C15B—C14B—H14C	109.1
N3A—C14A—H14B	109.3	N3B—C14B—H14D	109.1
C15A—C14A—H14B	109.3	C15B—C14B—H14D	109.1
H14A—C14A—H14B	108.0	H14C—C14B—H14D	107.8
C18A—C15A—C16A	117.19 (17)	C18B—C15B—C16B	116.3 (2)
C18A—C15A—C14A	124.70 (18)	C18B—C15B—C14B	125.5 (2)
C16A—C15A—C14A	118.03 (16)	C16B—C15B—C14B	118.1 (2)
O2A—C16A—C15A	121.25 (17)	O2B—C16B—C15B	121.0 (2)
O2A—C16A—C3A	121.91 (17)	O2B—C16B—C3B	120.6 (2)
C15A—C16A—C3A	116.83 (15)	C15B—C16B—C3B	118.40 (18)
N3A—C17A—H17A	109.5	N3B—C17B—H17D	109.5
N3A—C17A—H17B	109.5	N3B—C17B—H17E	109.5
H17A—C17A—H17B	109.5	H17D—C17B—H17E	109.5
N3A—C17A—H17C	109.5	N3B—C17B—H17F	109.5
H17A—C17A—H17C	109.5	H17D—C17B—H17F	109.5
H17B—C17A—H17C	109.5	H17E—C17B—H17F	109.5
C15A—C18A—C19A	128.15 (19)	C15B—C18B—C19B	131.1 (2)
C15A—C18A—H18A	115.9	C15B—C18B—H18B	114.4
C19A—C18A—H18A	115.9	C19B—C18B—H18B	114.4
C20A—C19A—C24A	117.9 (2)	C20B—C19B—C24B	117.8 (3)
C20A—C19A—C18A	123.1 (2)	C20B—C19B—C18B	124.6 (2)
C24A—C19A—C18A	119.00 (18)	C24B—C19B—C18B	117.6 (2)
C21A—C20A—C19A	121.0 (2)	C21B—C20B—C19B	120.9 (3)
C21A—C20A—H20A	119.5	C21B—C20B—H20B	119.5
C19A—C20A—H20A	119.5	C19B—C20B—H20B	119.5
C22A—C21A—C20A	118.8 (2)	C22B—C21B—C20B	118.6 (3)
C22A—C21A—H21A	120.6	C22B—C21B—H21B	120.7
C20A—C21A—H21A	120.6	C20B—C21B—H21B	120.7
C21A—C22A—F1A	119.3 (2)	C21B—C22B—C23B	122.9 (3)
C21A—C22A—C23A	122.7 (2)	C21B—C22B—F1B	118.3 (3)
F1A—C22A—C23A	118.0 (2)	C23B—C22B—F1B	118.9 (3)
C22A—C23A—C24A	118.2 (2)	C22B—C23B—C24B	118.6 (3)
C22A—C23A—H23A	120.9	C22B—C23B—H23B	120.7
C24A—C23A—H23A	120.9	C24B—C23B—H23B	120.7
C23A—C24A—C19A	121.3 (2)	C23B—C24B—C19B	121.1 (3)
C23A—C24A—H24A	119.4	C23B—C24B—H24B	119.4
C19A—C24A—H24A	119.4	C19B—C24B—H24B	119.4
C30A—C25A—C26A	117.4 (2)	C30B—C25B—C26B	117.6 (2)
C30A—C25A—C4A	119.1 (2)	C30B—C25B—C4B	119.1 (2)
C26A—C25A—C4A	123.46 (19)	C26B—C25B—C4B	123.3 (2)
C25A—C26A—C27A	121.3 (2)	C27B—C26B—C25B	121.1 (3)
C25A—C26A—H26A	119.3	C27B—C26B—H26B	119.4
C27A—C26A—H26A	119.3	C25B—C26B—H26B	119.4
C28A—C27A—C26A	118.7 (3)	C28B—C27B—C26B	118.8 (3)
C28A—C27A—H27A	120.7	C28B—C27B—H27B	120.6
C26A—C27A—H27A	120.7	C26B—C27B—H27B	120.6
C27A—C28A—F2A	118.9 (3)	C29B—C28B—C27B	122.7 (3)
C27A—C28A—C29A	122.3 (2)	C29B—C28B—F2B	118.8 (3)
F2A—C28A—C29A	118.9 (3)	C27B—C28B—F2B	118.5 (3)
C28A—C29A—C30A	118.3 (2)	C28B—C29B—C30B	118.1 (3)
C28A—C29A—H29A	120.9	C28B—C29B—H29B	120.9
C30A—C29A—H29A	120.9	C30B—C29B—H29B	120.9
C25A—C30A—C29A	122.1 (2)	C25B—C30B—C29B	121.6 (3)
C25A—C30A—H30A	119.0	C25B—C30B—H30B	119.2
C29A—C30A—H30A	119.0	C29B—C30B—H30B	119.2
			
C1A—N1A—C2A—C12A	−68.1 (2)	C1B—N1B—C2B—C12B	−37.9 (3)
C5A—N1A—C2A—C12A	160.99 (16)	C5B—N1B—C2B—C12B	92.2 (2)
C1A—N1A—C2A—C6A	42.5 (2)	C1B—N1B—C2B—C6B	73.4 (2)
C5A—N1A—C2A—C6A	−88.32 (18)	C5B—N1B—C2B—C6B	−156.44 (19)
C1A—N1A—C2A—C3A	164.59 (17)	C1B—N1B—C2B—C3B	−165.0 (2)
C5A—N1A—C2A—C3A	33.72 (18)	C5B—N1B—C2B—C3B	−34.8 (2)
N1A—C2A—C3A—C13A	−128.56 (16)	N1B—C2B—C3B—C13B	−113.50 (19)
C12A—C2A—C3A—C13A	110.22 (18)	C12B—C2B—C3B—C13B	119.6 (2)
C6A—C2A—C3A—C13A	−7.8 (2)	C6B—C2B—C3B—C13B	2.2 (3)
N1A—C2A—C3A—C16A	112.85 (15)	N1B—C2B—C3B—C16B	127.87 (17)
C12A—C2A—C3A—C16A	−8.4 (2)	C12B—C2B—C3B—C16B	1.0 (2)
C6A—C2A—C3A—C16A	−126.39 (16)	C6B—C2B—C3B—C16B	−116.40 (19)
N1A—C2A—C3A—C4A	−6.77 (17)	N1B—C2B—C3B—C4B	10.7 (2)
C12A—C2A—C3A—C4A	−128.00 (16)	C12B—C2B—C3B—C4B	−116.19 (19)
C6A—C2A—C3A—C4A	113.99 (16)	C6B—C2B—C3B—C4B	126.46 (18)
C13A—C3A—C4A—C25A	−27.4 (2)	C13B—C3B—C4B—C5B	138.6 (2)
C16A—C3A—C4A—C25A	93.34 (19)	C16B—C3B—C4B—C5B	−102.4 (2)
C2A—C3A—C4A—C25A	−149.25 (16)	C2B—C3B—C4B—C5B	15.5 (2)
C13A—C3A—C4A—C5A	101.28 (18)	C13B—C3B—C4B—C25B	13.3 (3)
C16A—C3A—C4A—C5A	−138.00 (16)	C16B—C3B—C4B—C25B	132.4 (2)
C2A—C3A—C4A—C5A	−20.59 (18)	C2B—C3B—C4B—C25B	−109.8 (2)
C1A—N1A—C5A—C4A	−178.87 (17)	C1B—N1B—C5B—C4B	176.8 (2)
C2A—N1A—C5A—C4A	−47.77 (19)	C2B—N1B—C5B—C4B	46.3 (2)
C25A—C4A—C5A—N1A	169.07 (16)	C25B—C4B—C5B—N1B	89.1 (2)
C3A—C4A—C5A—N1A	40.78 (18)	C3B—C4B—C5B—N1B	−36.6 (2)
C7A—N2A—C6A—O1A	−174.46 (18)	C7B—N2B—C6B—O1B	−173.1 (2)
C7A—N2A—C6A—C2A	−0.7 (2)	C7B—N2B—C6B—C2B	0.3 (2)
N1A—C2A—C6A—O1A	59.0 (2)	N1B—C2B—C6B—O1B	52.1 (3)
C12A—C2A—C6A—O1A	175.34 (19)	C12B—C2B—C6B—O1B	173.3 (2)
C3A—C2A—C6A—O1A	−56.5 (2)	C3B—C2B—C6B—O1B	−61.3 (3)
N1A—C2A—C6A—N2A	−114.63 (17)	N1B—C2B—C6B—N2B	−121.12 (19)
C12A—C2A—C6A—N2A	1.74 (19)	C12B—C2B—C6B—N2B	0.1 (2)
C3A—C2A—C6A—N2A	129.85 (16)	C3B—C2B—C6B—N2B	125.51 (19)
C6A—N2A—C7A—C8A	175.76 (19)	C6B—N2B—C7B—C8B	177.4 (2)
C6A—N2A—C7A—C12A	−0.8 (2)	C6B—N2B—C7B—C12B	−0.6 (2)
C12A—C7A—C8A—C9A	−1.7 (3)	C12B—C7B—C8B—C9B	−1.3 (3)
N2A—C7A—C8A—C9A	−177.8 (2)	N2B—C7B—C8B—C9B	−179.0 (2)
C7A—C8A—C9A—C10A	1.9 (3)	C7B—C8B—C9B—C10B	0.7 (4)
C8A—C9A—C10A—C11A	−0.2 (4)	C8B—C9B—C10B—C11B	1.2 (4)
C9A—C10A—C11A—C12A	−2.0 (3)	C9B—C10B—C11B—C12B	−2.5 (4)
C10A—C11A—C12A—C7A	2.2 (3)	C10B—C11B—C12B—C7B	1.8 (3)
C10A—C11A—C12A—C2A	175.2 (2)	C10B—C11B—C12B—C2B	178.7 (2)
C8A—C7A—C12A—C11A	−0.4 (3)	C8B—C7B—C12B—C11B	0.1 (3)
N2A—C7A—C12A—C11A	176.40 (17)	N2B—C7B—C12B—C11B	178.14 (19)
C8A—C7A—C12A—C2A	−174.80 (18)	C8B—C7B—C12B—C2B	−177.4 (2)
N2A—C7A—C12A—C2A	2.0 (2)	N2B—C7B—C12B—C2B	0.6 (2)
N1A—C2A—C12A—C11A	−58.0 (3)	N1B—C2B—C12B—C11B	−62.7 (3)
C6A—C2A—C12A—C11A	−175.8 (2)	C6B—C2B—C12B—C11B	−177.5 (2)
C3A—C2A—C12A—C11A	59.6 (3)	C3B—C2B—C12B—C11B	57.4 (3)
N1A—C2A—C12A—C7A	115.60 (17)	N1B—C2B—C12B—C7B	114.4 (2)
C6A—C2A—C12A—C7A	−2.23 (18)	C6B—C2B—C12B—C7B	−0.4 (2)
C3A—C2A—C12A—C7A	−126.83 (17)	C3B—C2B—C12B—C7B	−125.47 (18)
C14A—N3A—C13A—C3A	−71.46 (19)	C14B—N3B—C13B—C3B	−72.1 (2)
C17A—N3A—C13A—C3A	167.56 (17)	C17B—N3B—C13B—C3B	164.3 (2)
C16A—C3A—C13A—N3A	60.29 (18)	C16B—C3B—C13B—N3B	60.5 (2)
C4A—C3A—C13A—N3A	−176.04 (15)	C4B—C3B—C13B—N3B	−178.9 (2)
C2A—C3A—C13A—N3A	−59.48 (19)	C2B—C3B—C13B—N3B	−60.2 (2)
C17A—N3A—C14A—C15A	177.44 (18)	C13B—N3B—C14B—C15B	52.6 (3)
C13A—N3A—C14A—C15A	55.7 (2)	C17B—N3B—C14B—C15B	176.2 (2)
N3A—C14A—C15A—C18A	141.42 (19)	N3B—C14B—C15B—C18B	153.9 (2)
N3A—C14A—C15A—C16A	−35.1 (2)	N3B—C14B—C15B—C16B	−27.8 (3)
C18A—C15A—C16A—O2A	31.2 (3)	C18B—C15B—C16B—O2B	21.8 (3)
C14A—C15A—C16A—O2A	−152.0 (2)	C14B—C15B—C16B—O2B	−156.7 (2)
C18A—C15A—C16A—C3A	−147.87 (18)	C18B—C15B—C16B—C3B	−159.25 (19)
C14A—C15A—C16A—C3A	28.9 (2)	C14B—C15B—C16B—C3B	22.3 (3)
C13A—C3A—C16A—O2A	141.36 (19)	C13B—C3B—C16B—O2B	141.7 (2)
C4A—C3A—C16A—O2A	17.5 (3)	C4B—C3B—C16B—O2B	18.0 (3)
C2A—C3A—C16A—O2A	−96.6 (2)	C2B—C3B—C16B—O2B	−96.2 (2)
C13A—C3A—C16A—C15A	−39.5 (2)	C13B—C3B—C16B—C15B	−37.3 (2)
C4A—C3A—C16A—C15A	−163.39 (16)	C4B—C3B—C16B—C15B	−160.99 (19)
C2A—C3A—C16A—C15A	82.52 (19)	C2B—C3B—C16B—C15B	84.8 (2)
C16A—C15A—C18A—C19A	177.51 (19)	C16B—C15B—C18B—C19B	−178.4 (2)
C14A—C15A—C18A—C19A	1.0 (3)	C14B—C15B—C18B—C19B	−0.1 (4)
C15A—C18A—C19A—C20A	43.0 (3)	C15B—C18B—C19B—C20B	21.8 (4)
C15A—C18A—C19A—C24A	−138.4 (2)	C15B—C18B—C19B—C24B	−160.1 (2)
C24A—C19A—C20A—C21A	1.8 (4)	C24B—C19B—C20B—C21B	1.7 (4)
C18A—C19A—C20A—C21A	−179.5 (2)	C18B—C19B—C20B—C21B	179.8 (3)
C19A—C20A—C21A—C22A	0.5 (4)	C19B—C20B—C21B—C22B	−0.9 (4)
C20A—C21A—C22A—F1A	178.0 (3)	C20B—C21B—C22B—C23B	0.3 (5)
C20A—C21A—C22A—C23A	−1.6 (4)	C20B—C21B—C22B—F1B	−179.9 (3)
C21A—C22A—C23A—C24A	0.3 (4)	C21B—C22B—C23B—C24B	−0.5 (4)
F1A—C22A—C23A—C24A	−179.4 (2)	F1B—C22B—C23B—C24B	179.6 (2)
C22A—C23A—C24A—C19A	2.2 (4)	C22B—C23B—C24B—C19B	1.4 (4)
C20A—C19A—C24A—C23A	−3.2 (3)	C20B—C19B—C24B—C23B	−1.9 (4)
C18A—C19A—C24A—C23A	178.1 (2)	C18B—C19B—C24B—C23B	179.8 (2)
C5A—C4A—C25A—C30A	158.5 (2)	C5B—C4B—C25B—C30B	151.6 (2)
C3A—C4A—C25A—C30A	−79.4 (2)	C3B—C4B—C25B—C30B	−89.2 (3)
C5A—C4A—C25A—C26A	−20.5 (3)	C5B—C4B—C25B—C26B	−27.9 (3)
C3A—C4A—C25A—C26A	101.7 (3)	C3B—C4B—C25B—C26B	91.3 (3)
C30A—C25A—C26A—C27A	1.3 (4)	C30B—C25B—C26B—C27B	−2.5 (4)
C4A—C25A—C26A—C27A	−179.7 (3)	C4B—C25B—C26B—C27B	177.1 (2)
C25A—C26A—C27A—C28A	0.0 (5)	C25B—C26B—C27B—C28B	1.5 (4)
C26A—C27A—C28A—F2A	−180.0 (3)	C26B—C27B—C28B—C29B	0.9 (5)
C26A—C27A—C28A—C29A	−0.8 (5)	C26B—C27B—C28B—F2B	179.7 (3)
C27A—C28A—C29A—C30A	0.3 (5)	C27B—C28B—C29B—C30B	−2.0 (5)
F2A—C28A—C29A—C30A	179.4 (3)	F2B—C28B—C29B—C30B	179.2 (3)
C26A—C25A—C30A—C29A	−1.9 (4)	C26B—C25B—C30B—C29B	1.3 (4)
C4A—C25A—C30A—C29A	179.0 (2)	C4B—C25B—C30B—C29B	−178.2 (3)
C28A—C29A—C30A—C25A	1.2 (4)	C28B—C29B—C30B—C25B	0.8 (5)

### Hydrogen-bond geometry (Å, º)

**Table:**

*D*—H···*A*	*D*—H	H···*A*	*D*···*A*	*D*—H···*A*
N2*A*—H2*A*···O1*B*	0.86	2.11	2.959 (2)	169
N2*B*—H2*B*···O1*A*	0.86	2.14	2.984 (2)	169
C27*B*—H27*B*···O2*B*^i^	0.93	2.49	3.314 (3)	147

Symmetry code: (i) *x*, −*y*, *z*+1/2.
